# Body Composition Changes in the Immediate Peri-operative Period Following Total Joint Arthroplasty

**DOI:** 10.2478/joeb-2022-0007

**Published:** 2022-09-09

**Authors:** Michael C. Marinier, Ayobami S. Ogunsola, Jacob M. Elkins

**Affiliations:** 1 Department of Orthopedics and Rehabilitation, University of Iowa, Iowa City, IA USA

**Keywords:** Bioimpedance, arthroplasty, body composition, post-surgical changes

## Abstract

**Background:**

Bioimpedance analysis (BIA) is a readily available tool to assess body composition in a clinical setting. BIA has received little attention in orthopaedics and namely joint arthroplasty. This study aims to quantify changes in body composition in the immediate peri-operative period following total joint arthroplasty.

**Methods:**

Adults scheduled for elective total joint arthroplasty were recruited to participate. Patients underwent BIA scans in the immediate peri-operative period: pre-operative on their day of surgery, post-operative day 0, and post-operative day 1.

**Results:**

67 patients were enrolled to undergo BIA scans. Mean age was 62.64 ± 10.28 years old, and 49.2% were females. The all-supine cohort exhibited a 0.36 ± 0.61 kg increase in dry lean mass (*p* < 0.001) and 1.30 ± 2.14 kg increase in lean body mass on postoperative day 0 (*p* < 0.001). Patients received to 1.16 ± 0.58 kg of fluid mass, on average.

**Conclusion:**

BIA is a rapid, portable tool that allows for body composition analysis of an inpatient surgical population. This study demonstrated that BIA can detect net fluid changes and may approximate implant mass following total joint arthroplasty. This may aid surgeons in interpreting post-operative body composition changes.

## Introduction

Obesity is a well-known and increasing epidemic in the US. (1, 2) Obesity is stratified into increasing classes, I-III, by body mass index (BMI), which is defined by $\frac{weight\left(kg\right)}{height^{2}\left(m^{2}\right)}(2)$. This patient characteristic is relevant in many specialties including orthopaedic surgery. Specific to hip and knee arthroplasty, patients with BMI ≥ 40 kg/m^2^, also considered class III obesity, have traditionally been denied surgery (3, 4, 5, 6, 7) as morbid obesity has been shown to have increasing rates of postoperative complications such as delayed wound healing, periprosthetic joint infections, mechanical implant failure and increased pain (3, 8, 9, 10, 11, 12, 13, 14, 15).

Despite increasing evidence that BMI per se is an imperfect surrogate for complications, the use of BMI thresholds for surgical indications is actually increasing (16, 17, 18, 19). Furthermore, attempts at decreasing patients’ postoperative complications through simple body mass loss have been proven ineffectual and perhaps even counterproductive (7, 20, 21, 22). This contrasts attempts at identifying high-risk body composition metrics, such as percent body fat or pre-tibial fat deposition, which have been tied to increased post-operative complications (23, 24, 25, 26).

There have been investigations within orthopaedics examining various body composition imaging modalities including dual x-ray absorptiometry, CT scan, and full-body MRI, but there has not been widespread utilization (27, 28, 29, 30, 31). Furthermore, these technologies are not readily incorporated into outpatient or inpatient orthopaedic practice due to cost, exposure to ionizing radiation, time, and inconvenience.

Bioimpedance analysis (BIA) is an additional tool to analyze patients’ body composition (32, 33). This technology provides advantages over other modalities as it is more cost-effective, without exposure to ionizing radiation, and easily incorporated into clinical practice. Medical-grade, multi-frequency, stationary bioimpedance analyzers are available that can be employed in an outpatient clinical setting just as a conventional body-weight scale would be. Similarly, portable bioimpedance analyzers are available that can be transported readily to an inpatient unit for patients that are unable to readily stand.

There are limited studies examining BIA orthopaedics and even fewer in arthroplasty (23, 24, 33, 34). Furthermore, there exists a gap in knowledge regarding the immediate perioperative body composition changes that patients experience. In a recent case report, Wagner has found that metallic implants (titanium alloy nails) increase the fat-free mass (equivalent to lean body mass, LBM) as determined by BIA (35). To the study team’s knowledge, there have been no investigations examining these changes with arthroplasty implants.

This study aims to quantify the peri-operative body composition changes, namely LBM and dry lean mass (DLM), experienced by patients from the implantation of metallic prostheses following primary total joint arthroplasty (TJA).

## Materials and methods

Adult patients (>18 years of age) without a pacemaker scheduled to undergo primary TJA were recruited to undergo three multi-frequency BIA scans (InBody S10; InBody USA; Cerritos, CA USA). The InBody S10 utilizes six frequencies for impedance measurements (1, 5, 50, 250, 500, 1000 kHz) and three frequencies for reactance measurements (5, 50, 250 kHz). To complete the BIA exam, a tetrapolar eight-point electrode configuration is employed: two electrodes were positioned on each extremity at the bilateral ankles (medial and lateral), thumbs, and long fingers.

Two scans were conducted on the day of surgery: one pre-operative (DOS) and one post-operative (POD0). The third scan was conducted on post-operative day 1 (POD1). Height and weight measurements from the patient’s final clinic visit prior to surgery were utilized throughout the three scans. The scans were conducted in a seated or supine position, and while supine analysis was preferred, certain pre- and post-operative facilities did not have the ability to complete supine exams. Due to this, a subset of subjects underwent a mixture of seated and supine positioning across their three exams.

The peri-operative net volume change, implant model, and implant size for each subject were also recorded to account for perceived peri-operative changes. Net volume change was defined as the change in volume (e.g. fluid replacement, estimated blood loss, urine output, etc.) that patients experienced while they were under the anesthesia team’s care, which occurred in the pre-operative area, operating room, and post-anesthesia care unit. This value was then converted to mass using a fluid density approximation of 1.0 g/mL.

The primary outcome for this study was DLM changes. Secondary outcomes include changes in LBM. Descriptive statistics were used to analyze the distribution of variables and measures of central tendency (mean and standard deviation) were calculated for continuous variables. A paired t-test was used to estimate the difference between the means of DLM and LBM measured repeatedly in study subjects at DOS, POD0, and POD1. The level of significance for the paired t-test was set at 5% and a p-value less than 0.05 was statistically significant in the analysis. Statistical analysis was completed using SAS (SAS Institute; Cary, NC USA) and Microsoft Excel (Microsoft Corporation; Redmond, WA USA).

### Informed consent

Informed consent has been obtained from all individuals included in this study.

### Ethical approval

The research related to human use has been complied with all relevant national regulations, institutional policies and in accordance with the tenets of the Helsinki Declaration and has been approved by the authors’ institutional review board.

## Results

Over a ten-week enrollment period, 67 patients were prospectively enrolled to undergo BIA scans. Patient demographics and baseline values are shown in Table I.

**Table I:** Demography of patients and select pre-operative markers.

Variable (units)	Average (SD)
Age (years)	62.64 (10.28)
Male: Female (%)	50.8%: 49.2%
TKA: THA (%)	50.8%: 49.2%
Baseline BMI (kg/m^2^)	34.84 (7.56)
Baseline Weight (kg)	98.22 (25.81)
Baseline LBM (kg)	58.07 (14.41)
Baseline DLM (kg)	15.67 (3.67)

**Figure 1:**
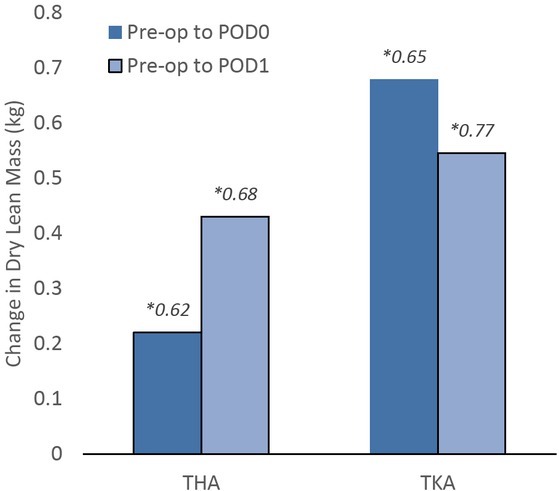
Comparison of means of change in dry lean mass (DLM) and procedure from the pre-operative scan on the day of surgery to postoperative day 0 (POD0) and day 1 (POD1) for total hip and knee arthroplasty. *Denotes standard deviation

Overall, patients gained 0.45 ± 0.67 kg of DLM and 1.67 ± 2.35 kg of LBM on POD0, which was, on average, 0.46% and 1.70% of their baseline body mass, respectively. Patients experienced 1158.85 ± 577.10 mL of net volume change in the peri-operative period (DOS to POD0), which is approximately 1.16 ± 0.58 kg of fluid mass. The three most commonly utilized knee and hip prostheses are included in Table IIa-c. Following the procedure (POD0), patients undergoing THA exhibited an increase of 0.22 ± 0.62 kg in DLM, while those undergoing TKA experienced 0.68 ± 0.65 kg DLM change (Figure 1).

**Table IIa:** The three most utilized metallic knee prostheses used during the study period. Note, femoral (F) and tibial (T) components were matched in all patients.

Rank (N)	Implant Brand and Model	Mean Size
1 (13)	MicroPort Evolution	F: 5.2; T: 5.2
2 (11)	DePuy Synthes Attune	F: 5.7; T: 5.0
3 (10)	Stryker Triathlon	F: 5.4; T: 4.9

**Table IIb:** The three most commonly implanted metallic acetabular cups.

Rank (N)	Implant Brand and Model	Mean Size (mm)
1 (12)	Zimmer-Biomet G7	55.2
2 (9)	DePuy Synthes Pinnacle	54.7
3 (8)	MicroPort Proctyl Prime	55.0

**Table IIc:** The three most commonly implanted metallic femoral stem prostheses.

Rank (N)	Implant Brand and Model	Mean Size
1 (10)	DePuy Synthes Actis	5.9
2 (8)	MicroPort Gladiator	4.6
3 (6)	Zimmer-Biomet Echo	12.2

**Figure 2:**
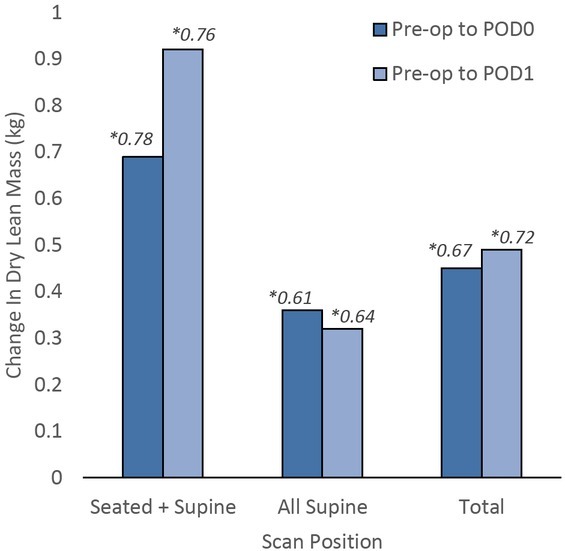
Comparison of means of change in dry lean mass (DLM) and scanning position from the pre-operative scan on the day of surgery to post-operative day 0 (POD0) and day 1 (POD1). *Denotes standard deviation

Forty-eight of the enrolled patients underwent exclusively supine scans. The all-supine cohort exhibited a 0.36 ± 0.61 kg change in DLM and 1.30 ± 2.14 kg change in LBM on post-operative day 0. Their changes in DLM and LBM on POD1 were 0.32 ± 0.64 kg and 1.16 ± 2.19 kg, respectively. Changes in DLM based on testing position are illustrated in Figure 2. In the entire sample, the difference between DLM and LBM, which estimates water mass change, was 1.22 kg from DOS to POD0.

## Discussion

The changes in LBM and DLM demonstrated significant (p<0.001) differences between pre-operative (DOS) and post-operative (POD0 and POD1) in both the supine and supine + seated cohorts. There was no difference between the two post-operative measurements (POD0 to POD1) for both DLM and LBM and in both positioning cohorts (Table III). Furthermore, comparison of DLM and LBM between the supine + seated and supine showed that there is no significant difference between these means regardless of the positioning.

**Table III:** The results of the paired t-test between changes in dry lean mass (DLM) and lean body mass (LBM) at different peri-operative time points. Separate analyses by positioning (supine and seated – left side; supine – right side) are included.

Supine and Seated N = 67		Mean (SD)	95% CL SD	t	*P*	Supine N = 47	Mean (SD)	95% CL SD	t	*P*
**LBM (kg)**	**DOS to POD0**	1.67 (2.35)	2.01, 2.83	5.81	<0.0001	**DOS to POD0**	1.30 (2.14)	1.78, 2.68	4.21	<0.001
	**DOS to POD1**	1.82 (2.55)	2.18, 3.08	5.82	<0.0001	**DOS to POD1**	1.16 (2.19)	1.82, 2.74	3.67	<0.001
	**POD0 to POD1**	0.15 (2.42)	2.06, 2.91	0.51	0.614	**POD0 to POD1**	-0.14 (2.51)	2.08, 3.14	-0.39	0.701
**DLM (kg)**	**DOS to POD0**	0.45 (0.67)	0.58, 0.81	5.53	<0.0001	**DOS to POD0**	0.36 (0.61	0.51, 0.76	4.08	<0.001
	**DOS to POD1**	0.49 (0.72)	0.62, 0.87	5.53	<0.0001	**DOS to POD1**	0.32 (0.64)	0.53, 0.79	3.41	<0.001
	**POD0 to POD1**	0.03 (0.70)	0.59, 0.84	0.38	0.705	**POD0 to POD1**	-0.04 (0.71)	0.59, 0.89	-0.42	0.679

Portable, multi-frequency BIA has been previously utilized to investigate body composition parameters for inpatient populations; however, to date there have been no specific analyses regarding total joint arthroplasty patients (36, 37, 38, 39). This study is the first to quantify body composition in the immediate perioperative period following total joint arthroplasty.

A 2014 report has shown that knee prostheses have an average mass of approximately 0.47 kg (40). This value is roughly 30% less than this study’s average DLM changes which likely indicates that changes in DLM are at least partially explained by the prosthesis mass. This increase in DLM was found to be a significant change between the preoperative scan and the two post-operative scans. There was

not however any difference in the two post-operative scans, which further corroborates that the entirety of the calculated changes occurs during the procedure.

These results build on the limited previous studies that demonstrate metallic implants lead to increased fat-free mass. (35) This change is likely secondary to the conductive nature of metallic prostheses, which more closely aligns with the conductivity of fat-free mass rather than fat mass. They also may guide surgeons utilizing BIA in their practice as the post-operative changes in body composition likely do not account for the mass of the metallic components. Thus, a gain in DLM secondary to metallic components may lead to inaccuracies when using BIA to measure body composition during follow-up.

Similar to the identified increases in DLM, there was a significant gain in LBM from the pre- to post-operative scan, and there was not a significant difference identified between LBM changes in the two post-operative scans. As DLM is simply LBM minus water mass, the difference in these body composition markers is likely the patients’ net fluid balance. This fluid increase is likely composed of extracellular fluid shifts such as post-operative swelling or surgical irrigation as well as intracellular fluid shifts resulting from medication administration. When this difference is compared to the average recorded net volume change, there is only a 0.06 kg discrepancy.

As for patient positioning with the portable bioimpedance analyzer, there was not a significant difference between the supine and supine + seated cohorts. This result implies that positioning during testing does not affect the outcome of the analysis, which may allow for flexibility in testing practices. As both cohorts produced significant differences in DLM and LBM, this result signifies that varying patient positioning may not sacrifice the utility of the results; however, following uniform study and practice protocols is favored.

This study is not without limitations. Foremost, as the portable bioimpedance analyzer used in this study does not have the ability to measure overall body mass, the body weight used was from patients’ final clinic visit prior to their surgical date. Therefore, changes in body weight from their last clinic visit to their surgical date are unaccounted for. In addition, this initial recorded body weight was utilized throughout the study’s three BIA exams rather than a new measurement per exam, which could affect the BIA results. Secondly, as discussed previously, body composition was not assessed in a uniform position. In addition, analysis of changes in body composition as it relates to net volume change were not completed on a patient-specific basis, but rather, these analyses were completed on a sample level. Finally, the conversion from volume to mass of the net fluid change was estimated using a standard conversion (1 g/mL) whereas each fluid including in this variable has a unique relative density (e.g. lactated ringers solution, blood, 5% albumin, urine, etc.).

Further directions of this work include analyzing patients undergoing revision and/or resection arthroplasty and capturing additional orthopaedic populations including those undergoing non-hip and non-knee arthroplasty, trauma, and spine procedures. Many of these studies are currently underway.

In conclusion, this study demonstrates that BIA can be incorporated into orthopaedic surgical practice to track the subtle changes that occur following the implantation of metallic prostheses. These findings will aid interpretation of peri-operative body composition changes.
